# MiR-199a-3p affects the multi-chemoresistance of osteosarcoma through targeting AK4

**DOI:** 10.1186/s12885-018-4460-0

**Published:** 2018-06-04

**Authors:** Wang Lei, Chen Yan, Jiang Ya, Dai Yong, Bian Yujun, Liu Kai

**Affiliations:** Department of orthopaedic surgery, the third people’s hospital of Hefei, Hefei, 230031 Anhui China

**Keywords:** Osteosarcoma, Multi-chemoresistance, miR-199a-3p, AK4

## Abstract

**Background:**

MicroRNAs (miRNAs) play vital roles in regulating various biological processes. The dysregulations of miRNAs may result in severe human diseases, including cancer.

**Methods:**

We performed the qRT-PCR, western blot and the luciferase reporter assays to test whether Adenylate Kinase 4 (AK4) is the target of miR-199a-3p. Up- or down-regulation of miR-199a-3p and/or the AK4 gene was done to detect their roles in OS multi-drug resistance using drug resistance profiling assays. We further predicted the putative signal pathway involved in the miR-199a-3p-mediated OS drug-resistance.

**Results:**

The AK4 gene is one of the targets of miR-199a-3p and negatively correlates with the effect of miR-199a-3p on OS drug-resistance. In addition, the activity of the NF-кB signaling pathway was drastically altered by the forced changes of the miR-199a-3p level in OS cells.

**Conclusions:**

Our data revealed that both miR-199a-3p and its target gene AK4 are reversely correlated with the OS drug resistance.

**Electronic supplementary material:**

The online version of this article (10.1186/s12885-018-4460-0) contains supplementary material, which is available to authorized users.

## Background

MiRNAs (miRNAs) are small non-coding RNAs which are recognized as vital and evolutionarily ancient components of gene regulation [1]. In the recent years, tremendous and growing studies have been focused on the role of mircoRNA (miRNA) in normal cellular as well as in disease processes, especially in cancer [2, 3]. The expression profiling of miRNAs has already been used in cancer clinics as diagnostic and prognostic biomarkers to assess tumor development [4]. The roles of various miRNAs were reported in different types of cancers, including breast, colon, gastric, lung, and prostate [5–7]. Specifically, one type of miRNA is also involved in different cancers. For instance, the accumulating studies showed that the dysregulation of miR-199a is found in various cancers, including hepatocellular carcinoma [8], ovarian cancer [9], renal cell carcinoma [10], osteosarcoma [11] and etc. [12, 13]. OS is the most common aggressive primary sarcoma of bone, which is usually occurred in children and adolescents [14, 15]. Metastatic OS usually has poor prognosis in response to the current chemotherapy mainly due to the chemoresistance. However, little is known about the underlying mechanism that governs the chemoresistance of OS. To address this issue, we put our effects on elucidating the relationship between miRNAs and drug-resistance, and have found that several miRNAs are involved in OS drug resistance by targeting different genes [16–19]. Notably, the previous report suggested that miR-199a-3p is down-regulated in OS [11]. However, whether miR-199a-3p is involved in the OS drug resistance is still unknown. In this study, using a systematic analysis in the multi-drug sensitive (G-292 and U2OS) and resistant (MNNG/HOS) OS cell lines, we found that miR-199a-3p inhibits multi-drug resistance of OS. We further revealed that miR-199a-3p targets the AK4 gene, which was reported to be involved in stress, drug resistance, malignant transformation in cancer [20–22]. Taken together, our findings provide a new mechanistic insight into OS drug resistance, which might give us hints for a rational design of the clinical therapy against OS.

## Methods

### Cell lines

G-292 (NO.CRL-1423), U2OS (NO.40342) and MNNG/HOS (NO.1547) were purchased from ATCC, and were cultured at 37 °C in DMEM medium (Biological Industries, Israel) supplemented with 10% fetal bovine serum (PAN) in a humidified incubator in an atmosphere containing 5% CO_2_. All cell lines were free of mycoplasma contamination.

### Real-time PCR analysis

Total RNA from cells was extracted in TRIzol Reagent. For detecting and quantifying the expression of specific miRNAs, RNA was reverse transcribed using a Bulge-Loop™ miRNA qRT-PCR Primer Set and quantified by SYBR Green-based real-time PCR analysis in the FTC-3000P PCR instrument. The Ct values of the target miRs were normalized to the Ct values of U6 RNA before quantification using the 2^−ΔΔ^ Ct method.

For the mRNA analysis, RNA (1 mg) was reverse-transcribed by using the PrimeScript RT reagent Kit with gDNA Eraser (Tiangen), the mRNA level of the AK4 gene was quantified using duplex-qRT-PCR analysis where the Taqman probes with different fluorescence for β-actin were used in the FTC-3000P. The following thermal settings were used: 95 °C for 20s followed by 40 cycles of 95 °C for 5 s and 60 °C for 30s. Using the 2^−ΔΔ^ Ct method, normalization to the β-actin level was performed before the relative levels of the target genes were compared. The sequences of the primers and probes used for the qRT-PCR analysis are:

- hAK4 F: 5′-CACTTCTTGCGGGAGAACATC-3′
  hAK4 R: 5′-CCAACTCGGACATCATTAGGC-3′
  hAK4 probe: 5′-FAM-CAGCACCGAAGTTGGTGAGATGGC-3′
- hACTB F: 5′-GCCCATCTACGAGGGGTATG-3′
  hACTB R: 5′-GAGGTAGTCAGTCAGGTCCCG-3′
  hACTB probe: 5′CY5-CCCCCATGCCATCCTGCGTC-3′

### Drug resistance profiling

The method of CCK8 assay according to the literature report [16, 19], the IC_50_ values with the no-drug control as the reference were calculated. The relative drug resistance was presented as the fold change in the IC_50_ of the cell lines relative to the lowest IC_50_.

### Cell transfection

The mimic, antagomir,agomir, siRNA, negative control (NC) and riboFECT CP transfection kit were supplied by Guangzhou Ribobio, China. And the reporter plasmids in Cignal Finder™ Pathway Reporter Arrays came from SABiosciences, USA. Transfection of both ribonucleic acid reagents or plasmids mentioned in this paper was performed according to the manufacturer’s instructions. The sequences used in this study are as follows:

si-AK4:

- GCCTAATGATGTCCGAGTT
- 5’-GCCUAAUGAUGUCCGAGUU dTdT-3′
- 3′-dTdT CGGAUUACUACAGGCUCAA-5’

### Luciferase reporter assay

Portion 5572–5579 of the AK4 3’-UTR combined with the target sequence for miR-199a-3p was cloned into the 3′ end of the luciferase-coding sequence of pEZX-MT01 to construct pEZX-MT01-luc-AK4 WT. The constructs were confirmed by DNA sequencing. The relative firefly luciferase activities of the UTR construct and pathway reporter constructs were analyzed as reported [23].

### Signaling pathway analysis

Constructs for the reporters of ten cancer signaling pathway, Wnt,Notch,p53/DNA damage, TGFβ, Cell cycle/pRb-E2F, NFκB, Myc/Max, Hypoxia, MAPK/ERK, MAPK/JNK and the Negative Control were obtained from SABiosciences and analyzed according to the manufacturer’s instructions. The analyzed as previously reported [19].

### Western blot analysis of protein

Cells were lysed with the lysis buffer and heated at 95 °C for 10 min. Anti-AK4 (AP20571a) was purchased from Abgent, anti-GAPDH (AM1020a) and HRP goat anti-mouse IgG antibody (LP1002a) were provided by Proteintech. The analyzed as previously reported [16].

### In vivo studies

Animal experiments were undertaken in accordance with the National Institutes of Health Guide for the Care and Use of Laboratory Animals. Animal research was approved by the biomedical ethics committee of Anhui Medical University. The animal study proposal was approved by the Institutional Animal Care and Use Committee (IACUC) of the University of Science and Technology of China (certificate number: LLSC20170464). All of the mouse experimental procedures were performed in accordance with the Regulations for the Administration of Affairs Concerning Experimental Animals approved by the State Council of People’s Republic of China.

BALB/c male nude mice of 3–4 weeks of age were used for this study. G-292 cells were embedded in BD Matrigel™ Matrix (Becton, USA) and subcutaneously injected into the two sites on the backs of mice, 1.5 × 10^7^ cells/site.The subsequent analyzed as previously reported [18, 19].

### Statistical analysis

The data are presented as the means, and the error bars indicate the S.D. All of the statistical analyses were performed using GraphPad Prism 5. A two-tailed Student’s *t*-test, a one-way analysis of variance was used to calculate statistical significance. *p* value of < 0.05 was considered significant.

## Results

### MiR-199a-3p promotes multi-drug resistance in OS cells

**Fig. 1:**
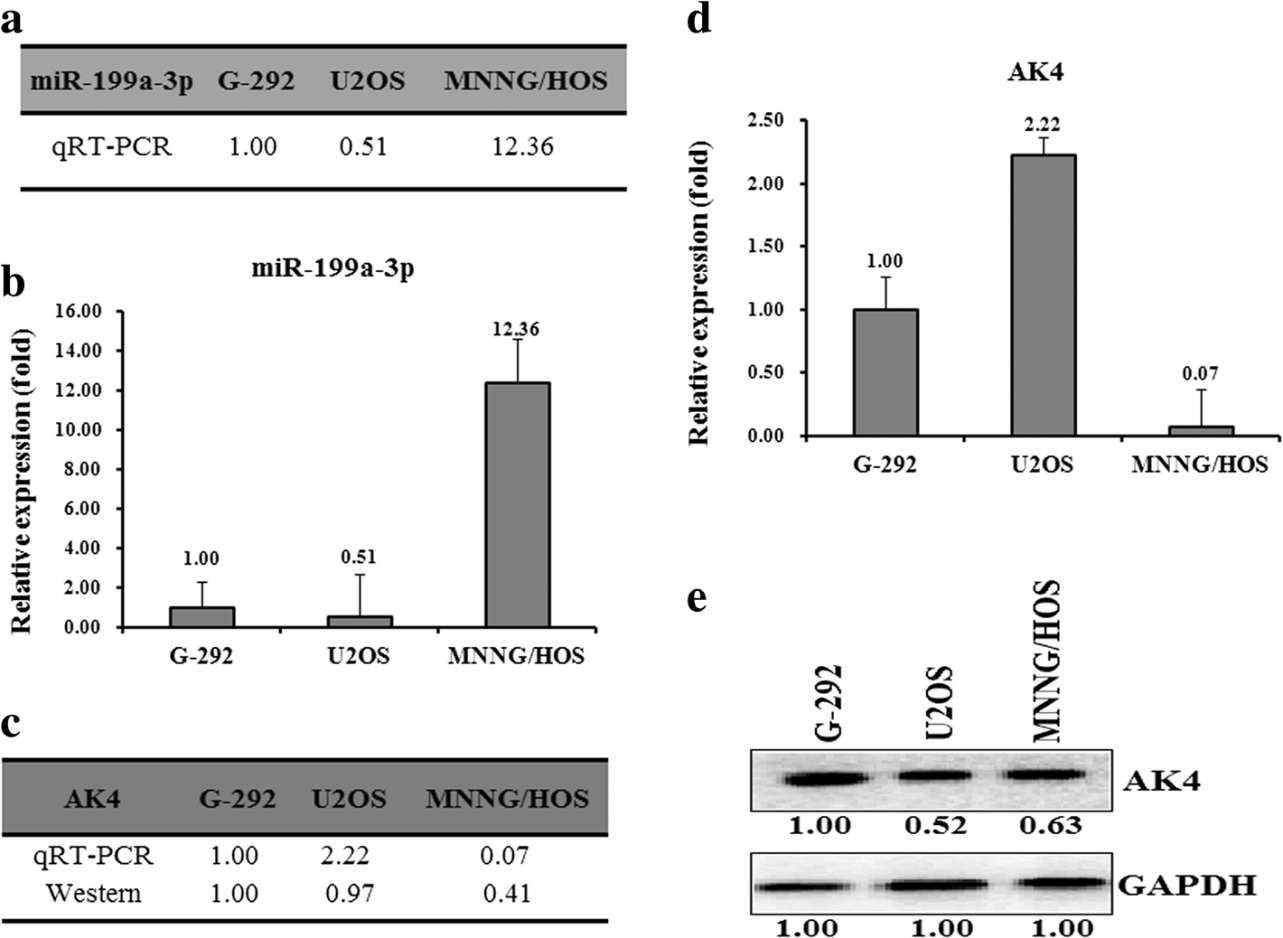
The relative miR-199a-3p level (fold) in G-292, U2OS and MNNG/HOS cell lines by qRT-PCR analyses is shown in table (**a**) and plot (**b**). The relative level (fold) of the AK4 gene in G-292, U2OS and MNNG/HOS cell lines was summarized in table (**c**), analyzed by qRT-PCR analyses in plot (**d**), and by western analysis (**e**)

To test whether miR-199a-3p is involved in OS drug resistance, we performed the drug resistance profiling assays using three commonly used OS cell lines (G-292, U2OS, and MNNG/HOS). We tested the IC_50_ values of these cell lines against the following drugs: cisplatin (CDDP), carboplatin (Carb), and doxorubicin (Dox). The results showed that G-292 possesses the lowest IC_50_ against all the three drugs, suggesting that G-292 is the most multi-drug sensitive cell line. Of note, the U2OS cells also showed a relatively low IC_50_ against the three drugs, resulting in a chemoresistance index of 6.54. By contrast, the MNNG/HOS cells have a relative drug resistance index of 74.16, indicating the feature of most drug-resistant characteristic (Additional file 1: Figure S1). To decipher the mechanistic insights that regulate the multi-drug resistance of OS, we selected miR-199a-3p as our target, which was previously identified to regulate OS drug resistance [24]. Consistently, as revealed by the qPCR assays, the expression of miR-199a-3p was relatively higher in MNNG/HOS cells than that in G-292 and U2OS cells, with the relative ratio of 12.36:1:0.56 for MNNG/HOS:G-292:U2OS (Fig. 1a and b). The results suggested that miR-199a-3p is involved in the multi-drug resistance of OS cells.

### AK4 is a target of miR-199a-3p in OS cells

MiRNAs usually down-regulate the target genes to fulfill their functions. We thus predicted the targets of miR-199a-3p using the following websites: TargetScan (http://www.targetscan.org/), miRDB (http://mirdb.org/miRDB/). Among them, we choose the AK4 gene as our target, which was previously found to be related to cancer drug resistance [25]. To further check the effect of the AK4 gene, we tested its expression at both mRNA and protein levels in the above OS cell lines. The results gave a higher expression level in G-292 and U2OS cells than that in MNNG/HOS cells (Fig. 1c, d and e). The ratio of mRNA and protein levels was 0.07:1.00:2.22 and 0.07:1.00:0.37 for MNNG/HOS:G-292:U2OS, respectively.

**Fig. 2:**
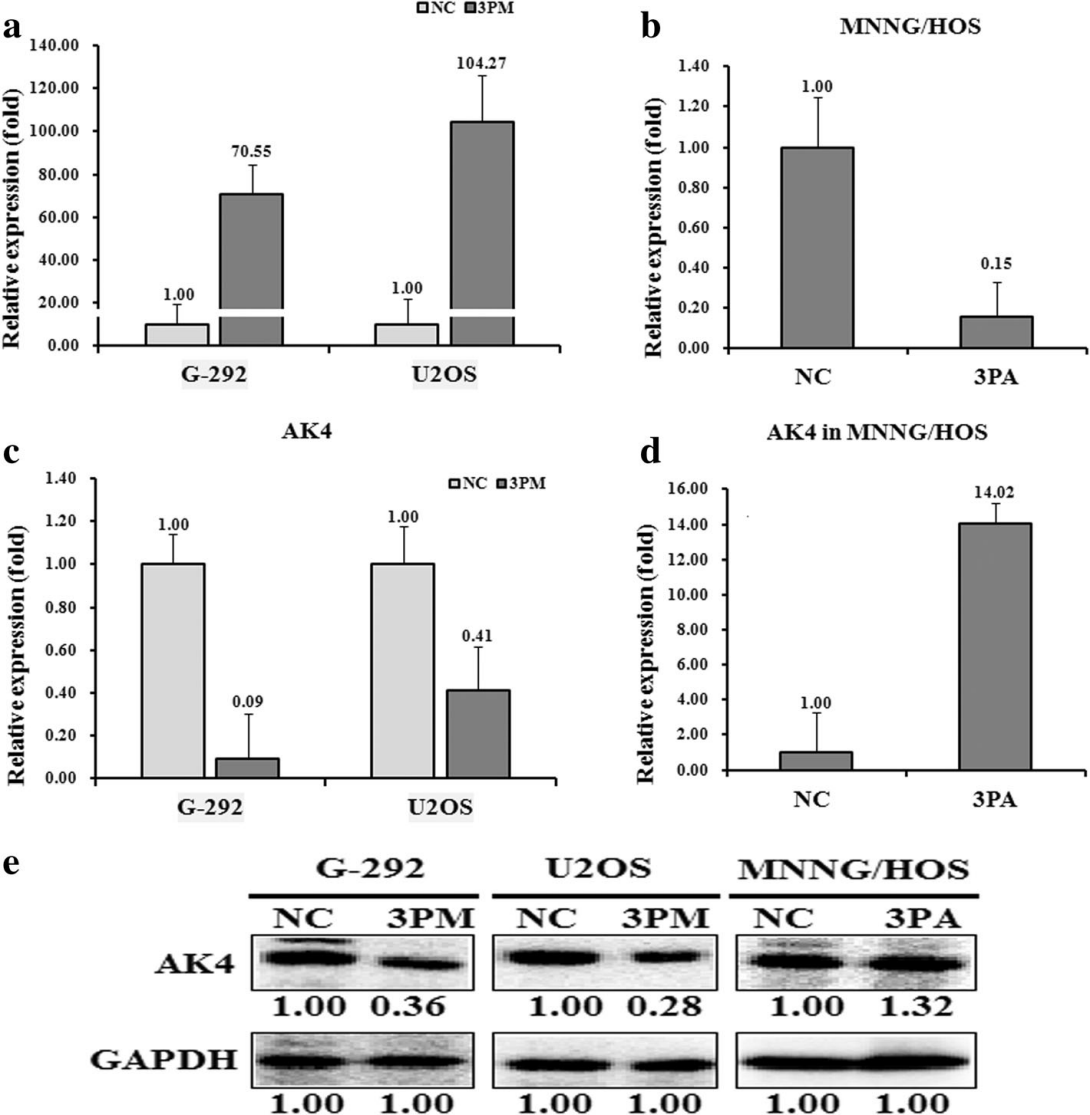
The level of miR-199a-3p (**a** and **b**). The AK4 mRNA (**c** and **d**) and protein (**e**) in the miR-199a-3p mimic (3 PM) transfected G-292 and U2OS cells and the miR-199a-3p antagomiR (3PA) transfected MNNG/HOS cells versus the negative control (NC), determined by qRT-PCR or Western analyses

We then determined the AK4 level in miR-199a-3p mimic-transfected G-292 and U2OS cells and antagomiR-transfected MNNG/HOS cells. Transfection of the miR-199a-3p mimic increased the miR-199a-3p level to approximately 70.55- and 104.27-fold, respectively, whereas the transfection of the miR-199a-3p antagomiR significantly down-regulated the miR-199a-3p level to 15% (Fig. 2a and b). Consistent with the changes of the miR-199a-3p level, transfection of the miR-199a-3p mimic down-regulated AK4 at both mRNA and protein levels compared to those with the NC-transfection (Fig. 2c, d and e). As expected, the miR-199a-3p antagomiR transfection increased the expression of AK4 in MNNG/HOS cells (Fig. 2c, d and e).

**Fig. 3:**
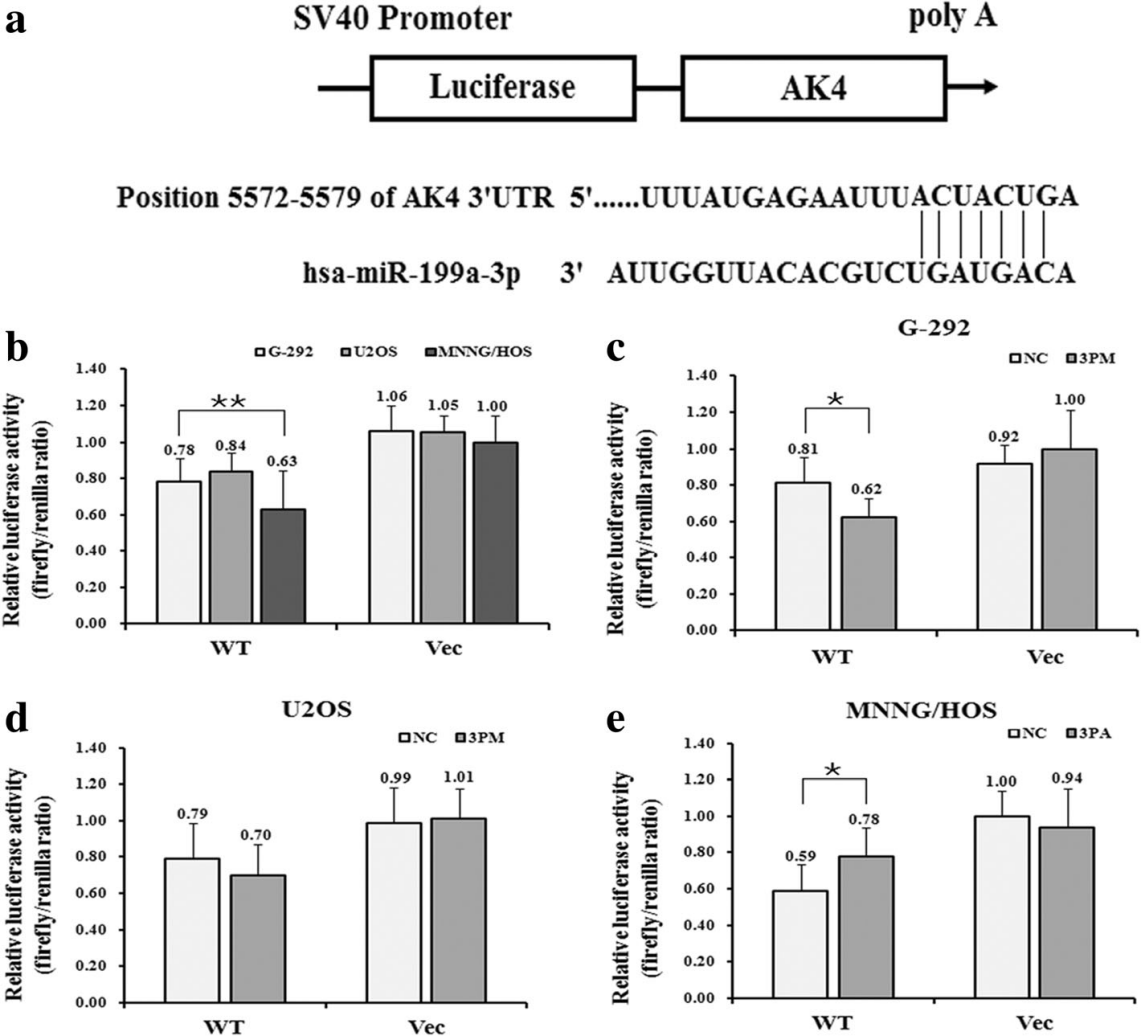
**a.** The sequences in UTR region of AK4 gene targeted by miR-199a-3p. **b-e**. The relative luciferase activity (fold) of the reporter with wild-type (WT) AK4-UTR or with no UTR (Vec) were determined in the miR-199a-3p mimic (in G-292 and U2OS) or antagomiR (in MNNG/HOS) or Mock transfected osteosarcoma cells. The renilla luciferase activity of a co-transfected control plasmid was used to control the transfection efficacy. The representative results from three independent experiments are shown. *P<0.05; **P<0.01 by Student’s *t*-test

Next, we constructed a reporter vector pGL3-AK4 UTR WT by the fusion of the 3′-untranslated region (UTR) of the AK4 gene harboring the putative binding site of miR-199a-3p with the *Renilla* luciferase gene (Fig. 3a). The construct was transfected into G-292, U2OS, and MNNG/HOS cells to determine whether AK4 is a target of miR-199a-3p. We found that pGL3-AK4-UTR WT led to a significantly higher luciferase activity in G-292 and U2OS cells than that in MNNG/HOS cells (Fig. 3b). Furthermore, the activity was increased in the antagomiR-transfected MNNG/HOS cells whereas was inhibited in the mimic-transfected G-292 and U2OS cells (Fig. 3c, d and e). Taken together, these results suggested that AK4 is indeed a target of miR-199a-3p.

### AK4 expression positively correlates with the drug resistance of OS cells

**Fig. 4:**
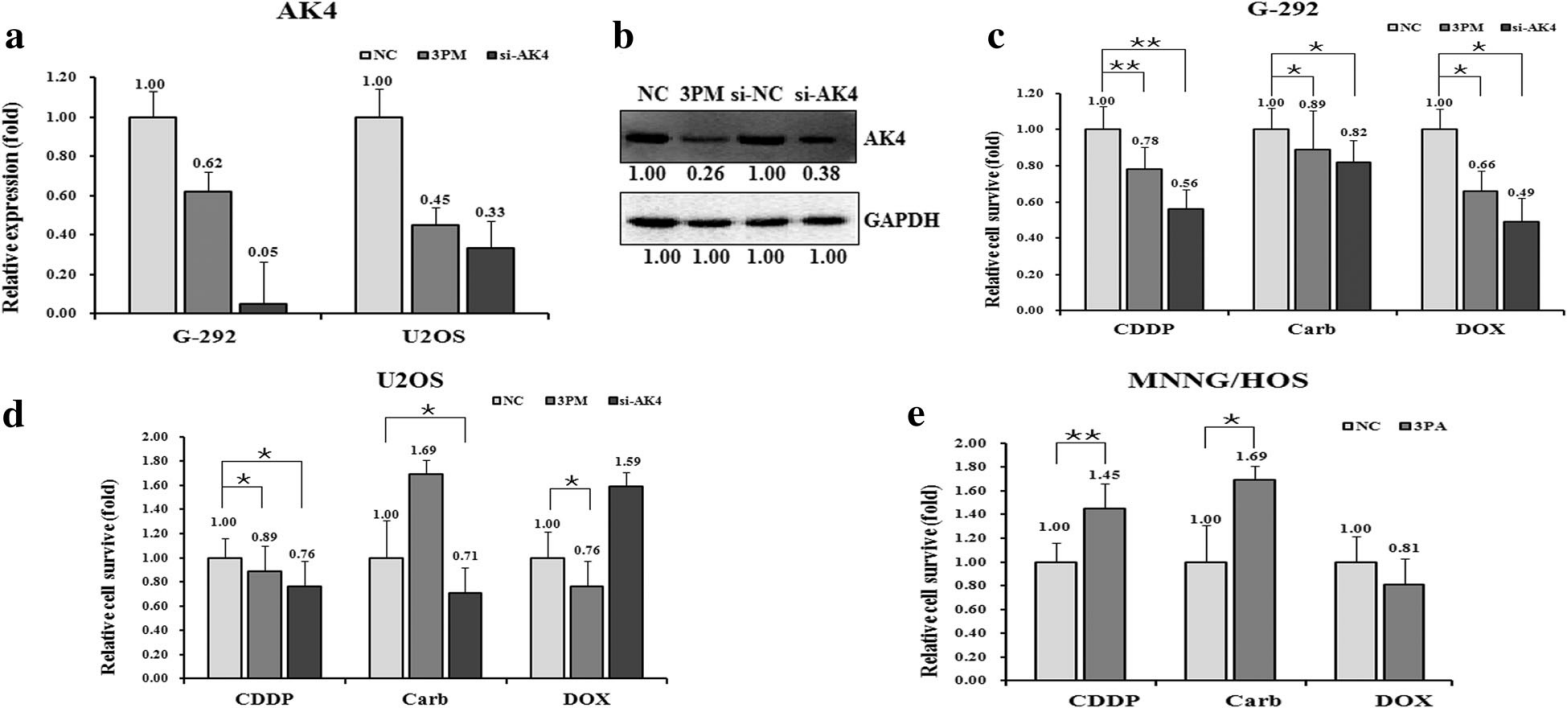
**a**. The levels by qRT-PCR of AK4 mRNAs in the mimic (3 PM) and siRNAs transfected G-292 and U2OS cells versus the NC. **b**. The AK4 protein level (western blot analysis) in the 3 PM and siRNAs transfected versus the NC transfected G-292 cells. **c**. The relative cell survival of the G-292 cells transfected by 3 PM and siRNAs over the NC transfected G-292 cells, 72 h after a treatment of the IC_50_ dosed drugs. **d**. The relative cell survival of the U2OS cells transfected by 3 PM and siRNAs over the NC transfected U2OS cells, 72 h after a treatment of the IC_50_ dosed drugs. **e**. The relative cell survival of the MNNG/HOS cells transfected by miR-199a-3p antagomiR (3PA) over the NC transfected MNNG/HOS cells, assayed at 72 h post-treatment of the IC_50_ dosed drugs. *P value < 0.05; **P value < 0.01

The functional relationship between miR-199a-3p and the AK4 gene in OS multi-drug resistance was then detected by comparing the effect on drug-triggered cell death in different OS cell lines. The transfection of either miR-199a-3p mimic or si-AK4 into G-292 or U2OS cells significantly decreased the AK4 level in both mRNA and protein levels (Fig. 4a and b). Accompanied with the decrease of AK4 level, the relative cell survival rate was somewhat dropped, indicating a decreased drug-resistance capability to all the three drugs, except U2OS to Carb and Dox (Fig. 4c and d). By contrast, the transfection of the miR-199a-3p antagomiR into MNNG/HOS cells increased the drug-resistance capability to all tested drugs, except MNNG/HOS to DOX (Fig. 4e). The results suggest that the expression of AK4 is positively correlated with the OS drug resistance.

### MiR-199a-3p regulates the activities of the NF-кB signaling pathway in the context of OS multi-drug resistance

**Fig. 5:**
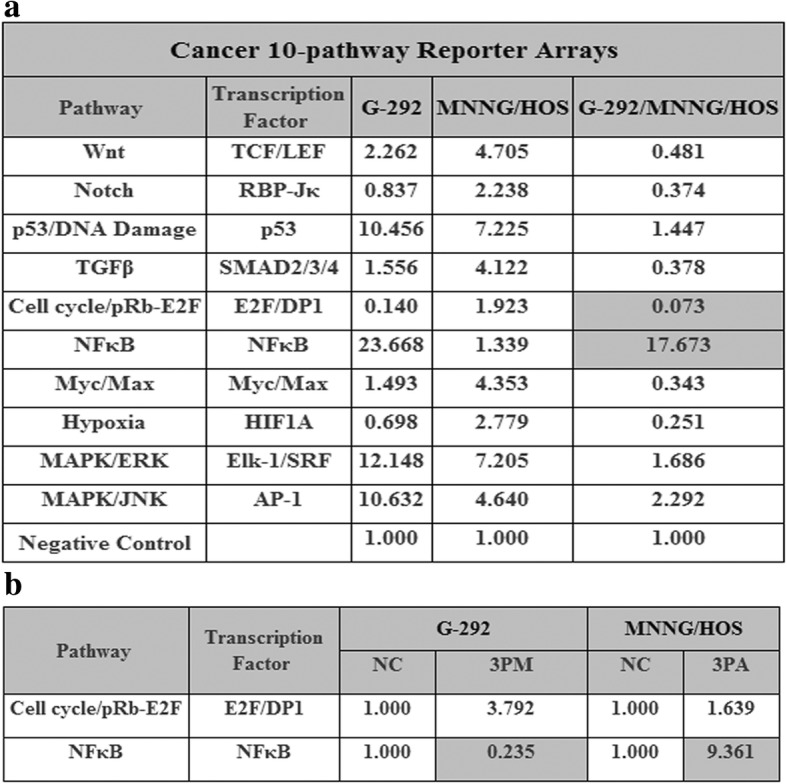
**a** The signaling pathways regulated by miR-199a-3p and their downstream genes. The relative activities (mean ± S.D.) of two pathways that differed by more than ten-fold between G-292 and MNNG/HOS cells. **b**. The relative activities of the pathways in the miR-199a-3p mimic (3 PM)-transfected versus NC-transfected G-292 cells as well as miR-199a-3p antagomiR (3PA)-transfected versus NC-transfected MNNG/HOS cells

To further elucidate the underlying mechanism of OS drug resistance mediated by miR-199a-3p, we measured the activities of ten cancer-related signaling pathways in both G-292 and MNNG/HOS cells (Fig. 5a). The results showed that the activities of NF-кB and Cell cycle/pRb-E2F were drastically differed by more than ten-fold between G-292 and MNNG/HOS cells (Fig. 5a), which indicates that they might play a role in OS drug resistance. The activity of NF-кB pathway is higher in G-292 cells, whereas that of the Cell cycle/pRb-E2F pathway is higher in MNNG/HOS cells (Fig. 5a). We then tested the expression level of these two pathways by forced changes in the miR-199a-3p level in both G-292 and MNNG/HOS cells. Upon the transfection of the miR-199a-3p mimic into G-292 cells, the activity of Cell cycle/pRb-E2F was increased accompanied by the elevation of the miR-199a-3p level. By contrast, the activity of NF-кB was repressed, which correlates well with the forced changes of the miR-199a-3p level in G-292 cells (Fig. 5b). We then transfected the miR-199a-3p antagomiR into MNNG/HOS cells to decrease of the miR-199a-3p level. As a result, the NF-кB pathway was up-regulated, which is in agreement with the negative effect of miR-199a-3p. However, the activity of Cell cycle/pRb-E2F is also increased which is contradictory to the results in G-292 cells. Overall, only the NF-кB pathway correlates well in the two cell lines, which indicates the involvement of the NF-кB pathway in the miR-199a-3p-mediated OS drug resistance.

### MiR-199a-3p inhibits both the growth and CDDP drug resistance of G-292-derived tumor xenografts in nude mice

**Fig. 6:**
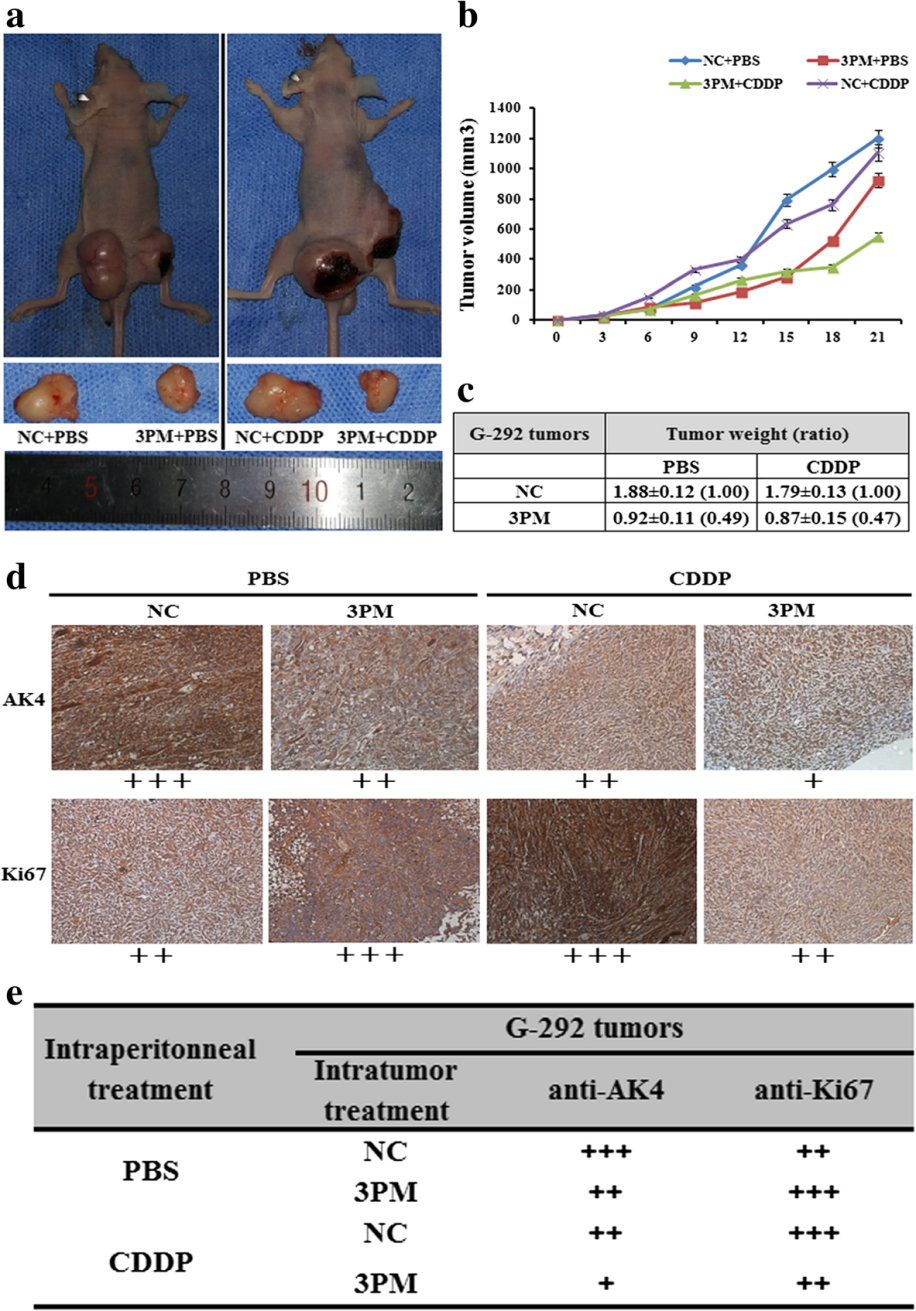
The miR-199a-3p’s effect on both the in vivo growth and CDDP chemoresistance of G-292 derived xenografts in nude mice. **b**. The tumor volume of the representative mice on the different time points. **c**. The list of the tumor weights on the day 25th. **d** and **e**. The levels of AK4 and Ki67 proteins in each group were determined by immunostaining and summarized in the table (Magnification: 200×)

The in vivo experiment was performed to test the role of miR-199a-3p in OS drug resistance. A model of G-292-derived tumor xenografts was subject to an intratumoral injection of miR-199a-3p agomiR, the scramble sequence control (Mock) or phosphate-buffered saline (PBS). After that the tumor mass was measured to compare the effect of miR-199a-3p on OS drug resistance with the intraperitoneal injection of PBS or CDDP. The results showed that an intratumoral injection of the miR-199a-3p agomiR decreased the tumor mass to about 49% (Fig. 6a, b and c), which suggests that miR-199a-3p is capable of inhibiting in vivo tumor growth. Moreover, the tumor weight of the miR-199a-3p agomiR transfected mice was much smaller than the control in the context of CDDP-treated group of G-292 cells (Fig. 6a, b and c).

Furthermore, we detected the level of AK4 and Ki67 (an indicator of tumor cell proliferation) by the immune-histological analysis in the tumor sections (Fig. 6d and e). The intratumoral injection of an miR-199a-3p agomiR into G-292 indeed led to a decrease of the AK4 level in the tumor sections (Fig. 6d and e), which further confirmed that miR-199a-3p has a negative effect on both the growth and drug resistance of OS cell-derived tumor xenografts in nude mice.

## Discussion

MiRNAs play vital roles in various biological processes such as proliferation, apoptosis and differentiation, via regulating gene expression at post-modification level [26]. Accumulating evidences have suggested that miR-199a-3p is involved in cancer biology [27, 28]. MiR-199a showed distinct expression profiles in several types of cancer [29, 30]. For instance, miR-199a-3p is downregulated in hepatocellular carcinoma, resulting in an increased sensitivity to doxorubicin-induced apoptosis [31]. Down-regulation of miR-199a-3p in cisplatin-resistant breast cancer is able to attenuate cisplatin resistance via regulating the mitochondrial transcription factor A [32]. All these studies indicated that miR-199a-3p may be involved in cancer chemotherapy resistance. In accordance with previous findings, here we showed that miR-199a-3p involves in OS multi-drug resistance. We systematically performed a series of functional assays and concluded that miR-199a-3p promotes the OS drug-resistance. We also found that the AK4 gene is a target of miR-199a-3p that positively correlates with the OS drug resistance (Additional file 2). Furthermore, we performed in vitro and in vivo experiments in cultured cells and tumor xenografts to address the roles of miR-199a-3p and AK4 in OS drug resistance.

AK4 was reported to be involved in the development of cancers, and is used as a potential therapeutic target for anticancer treatment. For example, the AK4 expression level could modulate the anti-cancer drug sensitivity through regulating mitochondrial activity [25]. Of note, a previous study found that AK4 promotes the metastasis of lung cancers by downregulating the transcription factor ATF3 [21]. In agreement with the previous findings, here we demonstrated that the expression level of AK4 is associated with the multi-drug resistance of OS cell lines, which might be regulated by miR-199a-3p. More investigations are needed to elucidate the relationship of miR-199a-3p and AK4 in regulating OS drug-resistance. In addition, the fine mechanism for the miR-199a-3p/AK4-mediated OS drug-resistance remains to be elucidated.

## Conclusions

In this work, we identified that miR-199a-3p could regulate OS multi-drug resistance, probably via controlling its target gene AK4. Our findings suggest that miR-199a-3p functions as a potential biomarker for treating OS chemoresistance.

## Additional files

Additional file 1: **Figure S1 A-C.** The IC_50_ of three indicated chemotherapeutics of three osteosarcoma cells. The percentage of the relative cell survival rates over the mock treatment was calculated and plotted against lg μM of drug. **D**. The relative IC_50_ (−fold) with the lowest IC_50_ (G-292 cell line) are presented in table. (TIF 5191 kb)

Additional file 2: The full-length gels of the relative level (fold) of the AK4 gene in G-292, U2OS and MNNG/HOS cells by western analyses in Figure S1E, the description of the data Please see 1. The full-length gels of level of AK4 protein levels in the miR-199a-3p mimic(3 PM)-transfected G-292 and U2OS cells and the miR-199a-3p antagomiR (3PA)-transfected MNNG/HOS cells versus the negative control (NC) cells in Figure S2E, as determined by western blot analyses, the description of the data Please see 2. The full-length gels of AK4 protein level by western blot analysis in the miR-199a-3p mimic (3 PM) and siRNA versus the NC-transfected G-292 cells, respectively in Figure S4B, the description of the data Please see 3. Tumor volume records detailed information in Figure S6B, the description of the data Please see 4. (PDF 167 kb)

## Abbreviations

AK4: Adenylate Kinase 4
Carb: Carboplatin
CDDP: Cisplatin
Dox: Doxorubicin
MiR: MicroRNA
OS: Osteosarcoma
UTR: Untranslated region
