# Supplementary figures and images for: MiR-199a-3p affects the multi-chemoresistance of osteosarcoma through targeting AK4
Source: BMC Cancer. 2018 Jun 4;18:631. doi: 10.1186/s12885-018-4460-0 (PMC5987492; doi:10.1186/s12885-018-4460-0)

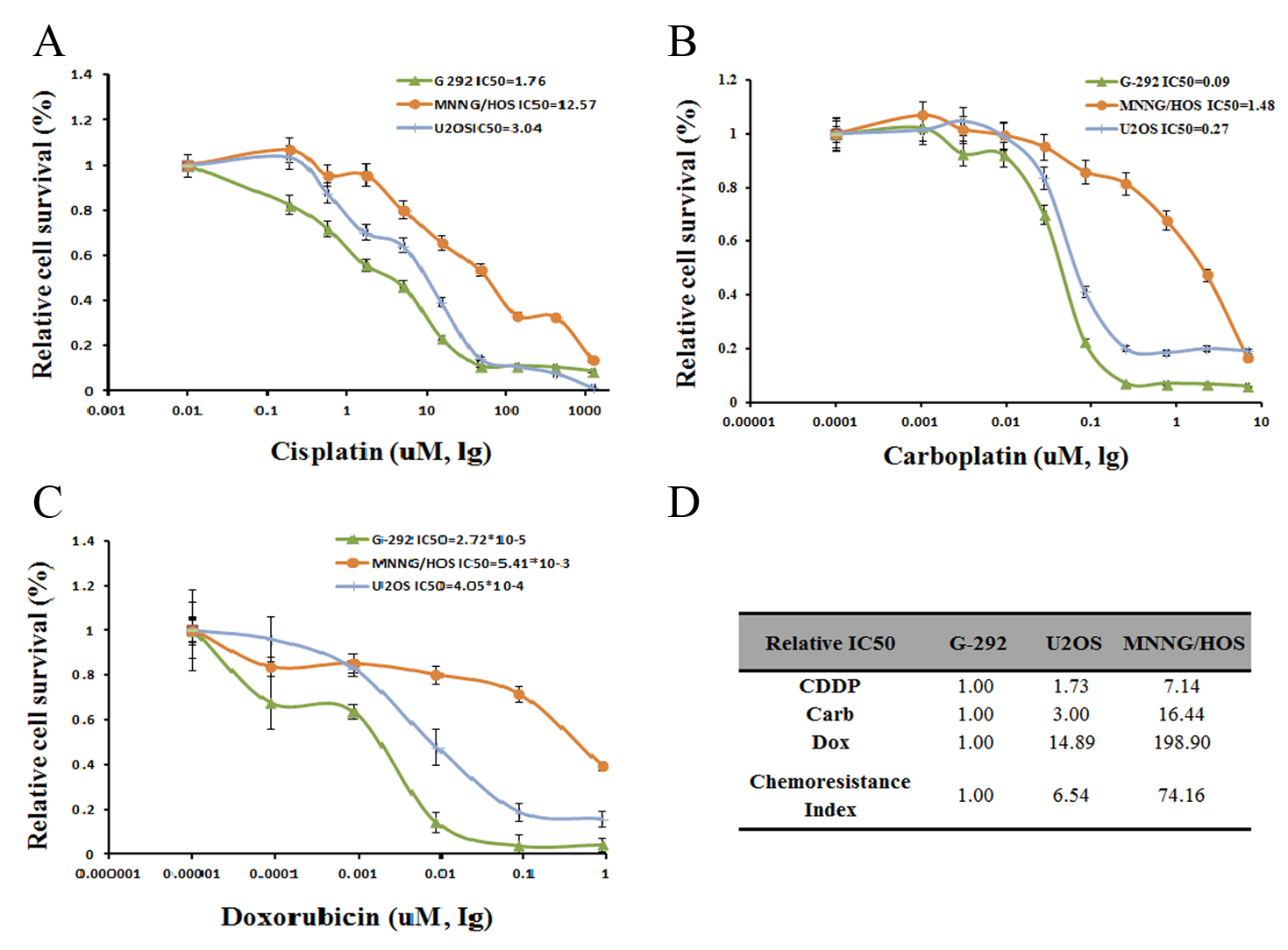

Supplement: Supplementary file 1 — Figure S1 A-C. The IC50 of three indicated chemotherapeutics of three osteosarcoma cells. The percentage of the relative cell survival rates over the mock treatment was calculated and plotted against lg μM of drug. D. The relative IC50 (−fold) with the lowest IC50 (G-292 cell line) are presented in table. (TIF 5191 kb) [file 12885_2018_4460_MOESM1_ESM.tif]
