# Supplementary material for: MiR-199a-3p affects the multi-chemoresistance of osteosarcoma through targeting AK4
Source: BMC Cancer. 2018 Jun 4;18:631. doi: 10.1186/s12885-018-4460-0 (PMC5987492; doi:10.1186/s12885-018-4460-0)
Supplement: Supplementary file 2 — The full-length gels of the relative level (fold) of the AK4 gene in G-292, U2OS and MNNG/HOS cells by western analyses in Figure S1E, the description of the data Please see 1. The full-length gels of level of AK4 protein levels in the miR-199a-3p mimic(3 PM)-transfected G-292 and U2OS cells and the miR-199a-3p antagomiR (3PA)-transfected MNNG/HOS cells versus the negative control (NC) cells in Figure S2E, as determined by western blot analyses, the description of the data Please see 2. The full-length gels of AK4 protein level by western blot analysis in the miR-199a-3p mimic (3 PM) and siRNA versus the NC-transfected G-292 cells, respectively in Figure S4B, the description of the data Please see 3. Tumor volume records detailed information in Figure S6B, the description of the data Please see 4. (PDF 167 kb) [file 12885_2018_4460_MOESM2_ESM.pdf]

The full-length gels of the western analyses used in the manuscript.

1. Figure 1E

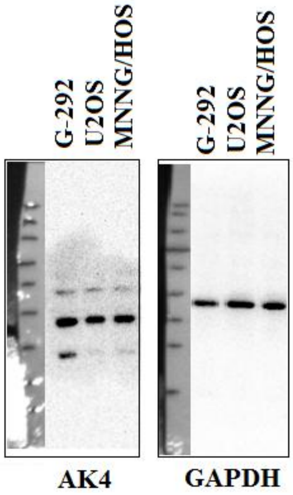


The full-length gels of the relative level (fold) of the AK4 gene in G-292, U2OS and MNNG/HOS cells by western analyses

2. Figure 2E

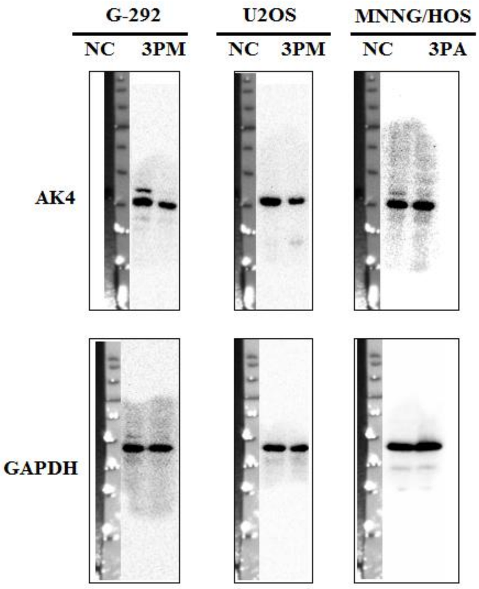


The full-length gels of level of AK4 protein levels in the miR-199a-3p mimic (3PM)-transfected G-292 and U2OS cells and the miR-199a-3p antagomiR (3PA)-transfected MNNG/HOS cells versus the negative control (NC) cells, as determined by western blot analyses.

3. Figure 4B

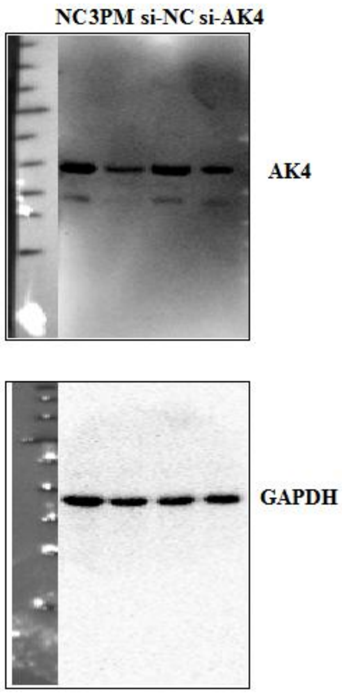


The full-length gels of AK4 protein level by western blot analysis in the miR-199a-3p mimic (3PM) and siRNA versus the NC-transfected G-292 cells, respectively.

4. Figure 6B

| No:20150706 | | Tumor volume record table | | | | | | | |
|---|---|---|---|---|---|---|---|---|---|
| Strain:Balb/c Nude | | | | | | | | | |
| Date:2015-07-18 | | | | | | | | | |
| Group | No. | 0 | 3 | 6 | 9 | 12 | 15 | 18 | 21 |
| NC+PBS | R | 48.54 | 69.00 | 98.04 | 203.09 | 242.35 | 369.53 | 673.59 | 977.99 |
| | L | 123.58 | 169.06 | 222.37 | 413.52 | 654.06 | 1392.61 | 1493.15 | 1595.65 |
| NC+CDDP | R | 87.34 | 94.35 | 180.70 | 229.15 | 361.56 | 434.46 | 631.97 | 896.46 |
| | L | 75.17 | 117.31 | 153.12 | 165.98 | 177.39 | 295.28 | 580.50 | 1112.00 |
| 3PM+CDDP | R | 109.78 | 139.11 | 283.79 | 470.52 | 593.42 | 712.82 | 817.19 | 1247.12 |
| | L | 164.47 | 270.04 | 409.28 | 657.82 | 812.56 | 1148.68 | 1580.04 | 1732.93 |
| 3PM+PBS | R | 51.42 | 82.70 | 113.18 | 193.59 | 271.30 | 341.82 | 367.34 | 585.68 |
| | L | 339.85 | 558.14 | 898.69 | 1044.44 | 1207.83 | 1279.41 | 1376.78 | 1579.25 |

Tumor volume records detailed information.
